# Temporal trends of *Shigella* outbreaks in the United States, 2009–2022

**DOI:** 10.3389/fpubh.2026.1740303

**Published:** 2026-02-12

**Authors:** Muhammad Rashid Bajwa, Charles Ayooluwa Adegbole, Rebecca Lee Smith

**Affiliations:** College of Veterinary Medicine, University of Illinois Urbana-Champaign, Urbana, IL, United States

**Keywords:** disease outbreaks, joinpoint regression, NORS, retrospective studies, *Shigella*

## Abstract

**Background:**

*Shigella* infections remain a significant public health threat in the United States because of their diverse outbreak patterns and high disease impact, particularly among young children. This study aimed to investigate the trends in *Shigella* outbreak incidence and demographic patterns by age and sex from 2009 to 2022 in order to inform targeted prevention strategies.

**Methods:**

We performed a retrospective observational analysis of 1,208 *Shigella* outbreak surveillance data sourced from the Centers for Disease Control and Prevention (CDC) via the National Outbreak Reporting System (NORS). The incidence rates per 1 million population–years were calculated overall and by age and sex. Descriptive statistics were used to summarize the data, and joinpoint regression with 95% confidence intervals was used to identify trends.

**Results:**

The outbreak incidence rate was 0.27 (95% CI: 0.01–0.84) per 1 million population–years in 2009, peaking at 0.74 (95% CI: 0.65–0.84) in 2016 and declining to 0.03 (95% CI: 0.01–0.05) by 2022. Children aged 1–4 years had the highest incidence, and females had a slightly higher incidence rate than males, as indicated in outbreak–associated case data. Joinpoint incidence trends showed an annual percent change (APC) of 25.12% (95% CI: 11.47–53.76, *p* = 0.001) from 2009 to 2015, followed by a decline with an APC of −38.91% (95% CI: −60.07 to −29.57, *p* < 0.001). The outbreaks were predominantly person–to–person transmission, with the highest frequency occurring in childcare settings.

**Conclusion:**

These findings underscore the need for targeted prevention strategies, such as ongoing hygiene education and sanitation enhancements in childcare, as well as reminders about *Shigella* prevention in shared educational facilities before the fall semester.

## 1 Introduction

Shigellosis, caused by *Shigella* spp., is among the leading causes of diarrheal infections worldwide, with an estimated 80–165 million cases and 600,000 deaths annually (1). In the United States (US), *Shigella* causes an estimated 500,000 cases, 100,000 hospitalizations, and 500 deaths each year (2). *Shigella* spp. are highly infectious (3), and are transmitted primarily through the fecal–oral route, including person–to–person contact, and via contaminated food, water, and the environment (4). The antimicrobial resistance of *Shigella* to third–generation cephalosporins is increasing, with a more than doubling from 5% in 2020 to 12.3% in 2022, alongside recent findings of high resistance to azithromycin (42%) and ciprofloxacin (18%) (5). The high incidence and development of multidrug resistance make *Shigella* spp. a critical public health and sustainability concern in the food industry.

Shigellosis was the third most common cause of enteric outbreaks in the US (after Norovirus and *Salmonella*) (6) and the second most common outbreak etiology among school and childcare settings (after Norovirus) from 2009 to 2019 (7). Although outbreaks among unhoused individuals and men who have sex with men (MSM) have also been reported (2, 8), children aged 1–4 years consistently exhibited the highest incidence rates during 2009–2018 in the US (9). This age concentration underscores the need to better understand transmission dynamics in community and childcare environments.

Inadequate sanitation, overcrowding, and poor hygiene are significant modifiable risk factors that disproportionately contribute to *Shigella* transmission in socioeconomically disadvantaged communities (10). Environmental factors, particularly seasonal temperature fluctuations, have also been shown to influence *Shigella* outbreak patterns, with higher incidence rates typically observed during warmer periods (8, 11, 12). These contextual factors underscore the importance of assessing long-term trends and their underlying drivers to inform targeted prevention efforts.

Previous studies have focused on the 10 US states within the Foodborne Diseases Active Surveillance Network (FoodNet), describing incidence rates, demographics (9, 13–17), and enteric outbreaks (2009–2018) (6); waterborne disease outbreaks associated with drinking water (18); untreated recreational water outbreaks from three US states (2008–2019) (19), acute gastroenteritis outbreaks by transmission mode (2009–2013) (20), and acute gastroenteritis outbreaks by person–to–person contact (2009–2010) (21). However, these previous studies have not fully addressed the long–term patterns underlying *Shigella* outbreaks. Despite the importance of *Shigella* outbreaks to public health in the US, comprehensive, long-term (14-year) studies describing the epidemiology, demographics, outbreak rates, outbreak-associated incidence, and temporal trends in the US as a whole are lacking.

Our study aimed to (1) assess the epidemiological characteristics of *Shigella* outbreaks between 2009 and 2022, including demographics, outbreak settings, and transmission modes, and (2) identify and quantify changes in *Shigella* outbreaks and outbreak–associated case incidence rates (IRs) during the study period.

Our study employed joinpoint regression analysis, a widely used statistical method for detecting and characterizing significant changes in temporal trends across various health indicators (22). Applying joinpoint regression to analyze national *Shigella* outbreak surveillance data from the National Outbreak Reporting System (NORS) represents a novel approach for examining trends in outbreak rates and associated case incidences.

## 2 Methods

### 2.1 Data sources and study design

We obtained *Shigella* spp. outbreak data from the Centers for Disease Control and Prevention’s (CDC) National Outbreak Reporting System (NORS) via a data request. The dataset included *Shigella* outbreaks with first illness onset dates from January 1, 2009, to December 31, 2022. NORS is a web-based central repository for outbreak data compiled from investigations and voluntary reports by territorial, local, and state health departments in the US Since 2009, this database has been the standard for all enteric disease outbreaks, including those caused by *Shigella* spp. in the US (23). An outbreak is defined as two or more cases of similar illness with a shared exposure (23). Outbreaks with at least two primary illnesses with confirmed or suspected *Shigella* etiology were selected.

The epidemiological characteristics of each outbreak, including geographic location, mode of transmission, date of onset of the first illness, and incubation period, were extracted from the NORS database. Moreover, the dataset included aggregated data on cases of illness within the outbreaks, including outcomes (hospitalizations, emergency room visits, and deaths), sex (male, female, and unknown), case status (confirmed, probable, and estimated total), and age group (<1, 1–4, 5–9, 10–19, 20–49, 50–74, and ≥75 years); these data were provided in a relational format. In NORS, outbreak-associated cases are classified as confirmed, probable, and estimated. For incidence calculations, we used the CDC-estimated case counts, which incorporate all reported case classifications. We selected outbreaks with at least two primary illnesses with confirmed or suspected *Shigella* etiology, consistent with the standard NORS definition that had already been applied in the finalized CDC dataset. This approach allowed us to include all outbreaks, including those with small case counts or from states with only a single reported outbreak. We used annual population data from 48 contiguous states and the District of Columbia (DC) from the US Census Bureau (24) for the statistical analysis.

Exposure-state assignments followed the CDC-defined Exposure State variable in the NORS dataset, with no recoding or modification by the authors. For outbreaks with a single exposure state, the Exposure State field identifies where exposure occurred, even when cases reside in multiple states; the corresponding Exposure State Count field provides the number of cases associated with that state. For outbreaks involving multiple exposure states, both fields remain blank, and the separate States table documents all implicated states, whether exposure or residency states. We used the exposure-state assignments exactly as provided by CDC in the finalized dataset of 1,226 outbreaks reported from 2009 to 2022.

### 2.2 Statistical analysis

#### 2.2.1 Descriptive analysis

We calculated the annual *Shigella* outbreak incidence rates per 1 million population–years, overall and by transmission mode, from 2009 to 2022, by dividing the total number of outbreaks each year by that year’s population and multiplying by 1 million. We also calculated outbreak–associated case-wise incidence rates per 1 million population–years overall and by sex and age group. The 95% confidence intervals (CIs) for incidence rates were calculated via the Poisson distribution. For the age- and sex-stratified incidence analyses, we included only outbreak-associated cases with known age or sex information. Cases with missing or unspecified age or sex were excluded solely from the corresponding stratified calculations; however, the outbreaks to which these cases belonged remained in the overall dataset. We also summarized the epidemiological characteristics of the outbreaks and associated cases, including reported *Shigella* species, symptoms, exposure settings, and transmission modes, by year and by month to investigate temporal and seasonal trends in outbreak characteristics.

We performed all the data cleaning, statistical analysis, summaries, and visualizations using the R environment version 4.5.0 (25) within RStudio IDE version 2025.5.1.513 (26).

#### 2.2.2 Joinpoint regression analysis

Our study employed Joinpoint Regression Analysis to analyze trends in *Shigella* outbreak data from 2009 to 2022, using annual aggregations of four population-standardized datasets: outbreak counts, outbreak-associated case counts, sex-specific case counts, and age-specific case counts, with rates standardized per 1 million population years. We used the Joinpoint Regression Program (version 5.4.0) (27) to identify change points in trends, with a significance level of *α* < 0.05, and assumed Poisson variance to calculate the Annual Percent Change (APC). Given our 14 annual data points (2009–2022), we allowed a maximum of two joinpoints via the Gris Search method, which is consistent with the software’s recommendations for model stability (22). The Weighted Bayesian Information Criterion (WBIC) (28) was used to determine the optimal number of joinpoints by balancing model fit and complexity. The empirical quantile method was used to calculate 95% confidence intervals for the Annual Percent Change (APC) and joinpoint locations. The Average Annual Percent Change (AAPC) was estimated to summarize the overall trend across the study period (2009–2022) (22).

## 3 Results

### 3.1 Outbreak incidence

We extracted data from 1,226 *Shigella* outbreaks reported to NORS with a first illness onset between 2009 and 2022. For this and future state-level spatial analyses, we included only outbreaks from the 48 contiguous states and the District of Columbia (DC), resulting in 1,208 outbreaks after excluding Alaska and Hawaii to maintain geographic contiguity. The incidence of *Shigella* outbreaks in the United States decreased from 0.27 (95% CI: 0.21–0.33) in 2009 to 0.03 (95% CI: 0.01–0.05) in 2022, with a peak of 0.74 (95% CI: 0.65–0.84) per million population–years in 2016. After stabilizing at approximately 0.22–0.29 post–2016, outbreak rates reached their lowest level during the COVID–19 pandemic years (0.09 in 2020 and 0.04 in 2021), remaining at 0.03 per million population–years in 2022.

**Figure 1:**
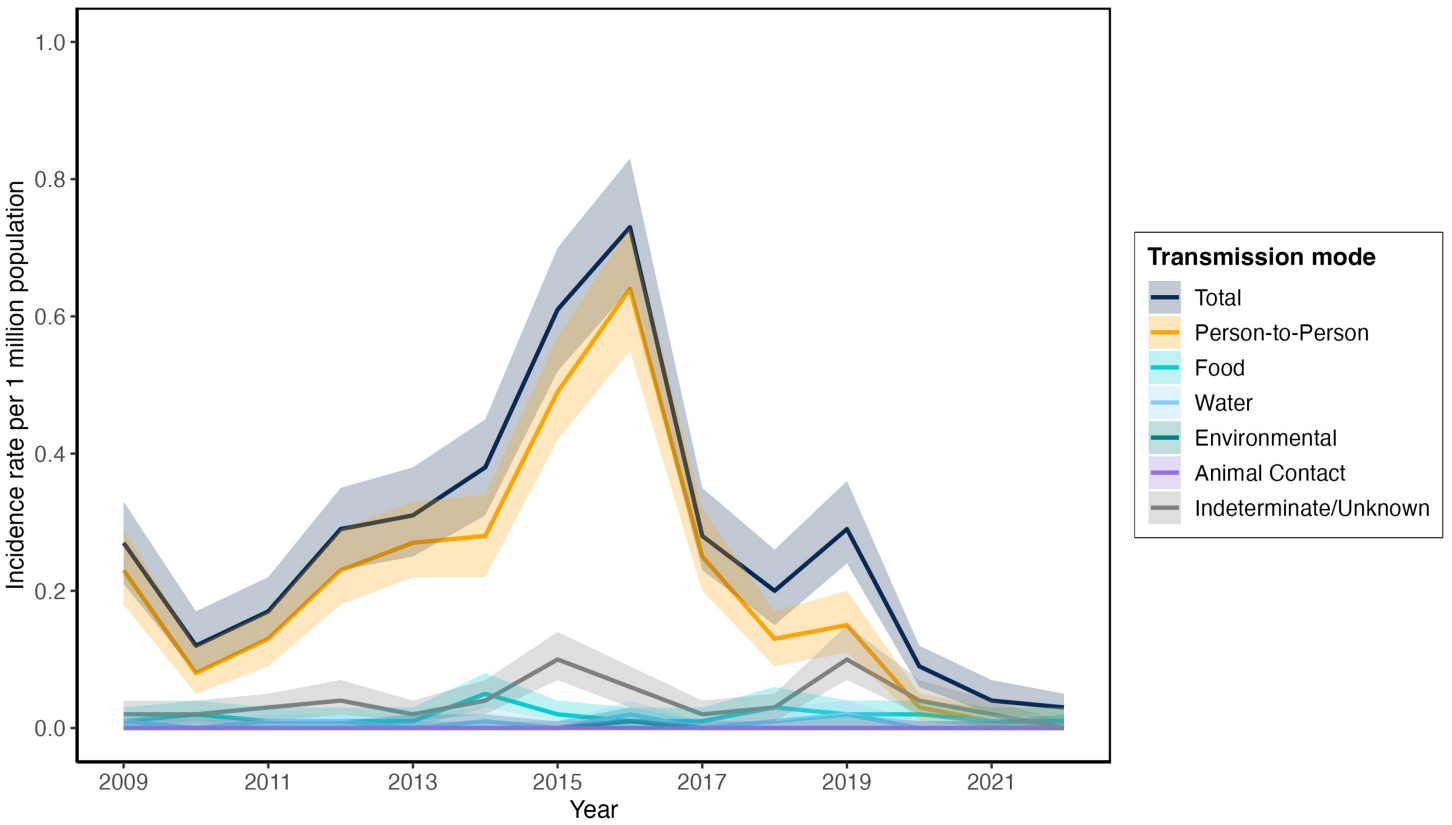
*Shigella* outbreak incidence rate per 1 million population by transmission mode in the United States, 2009–2022.

In the study population, person–to–person transmission was the most common primary mode, with 0.49–0.64 outbreaks per million population years in the high–activity years of 2015–2016 and 0.13–0.25 outbreaks per million population years in the lower–activity years. The only exception to this was for 2020–2022, during which lower person–to–person transmission corresponded to the first two years of the COVID–19 pandemic (Figure 1; Supplementary Table S6).

### 3.2 Outbreak–associated case incidence

**Figure 2:**
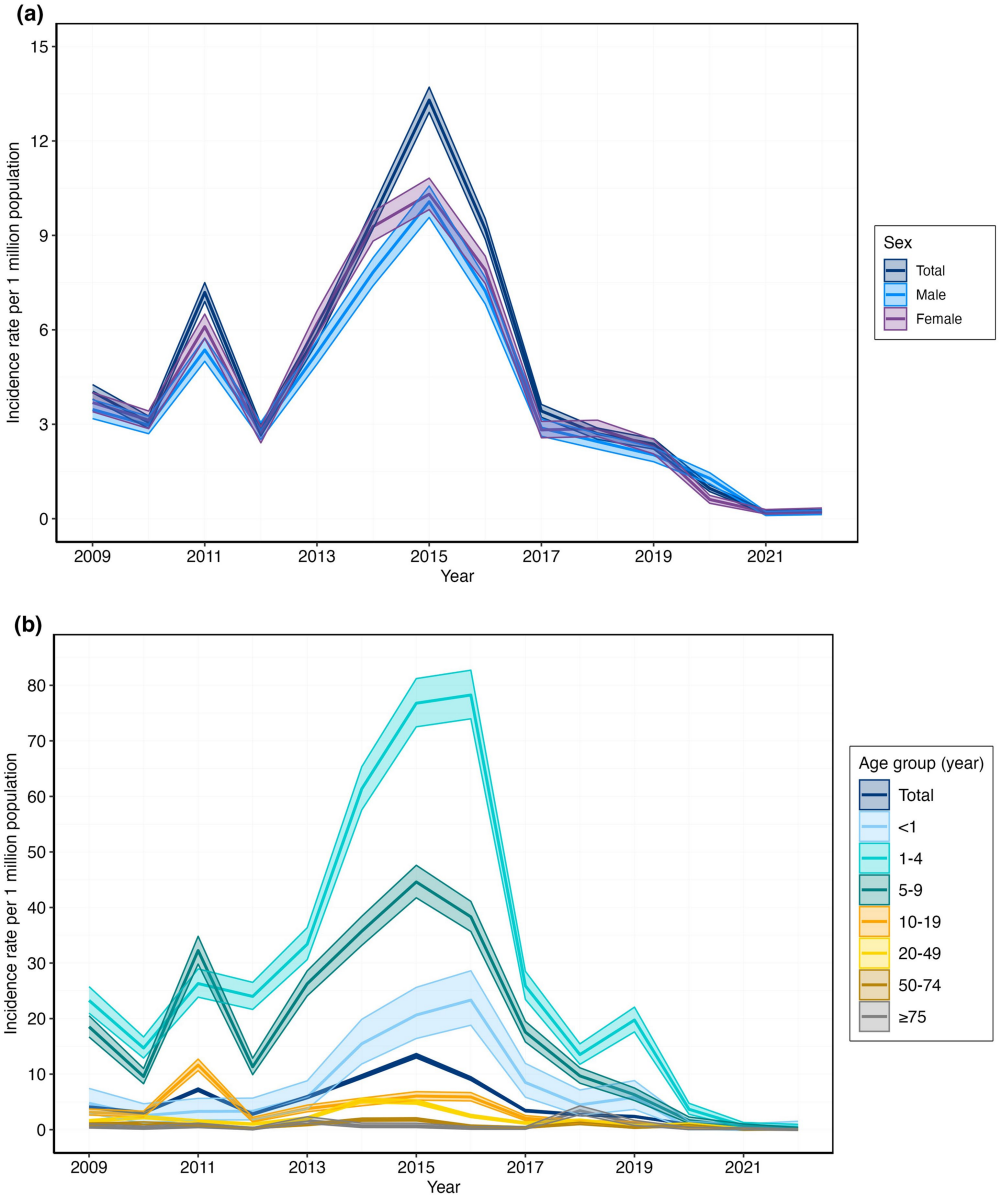
*Shigella* outbreak–associated case incidence rate per 1 million population in the United States, 2009–2022: **(a)** Incidence rate by sex; **(b)** incidence rate by age group.

The *Shigella* outbreak–associated case incidence in the United States followed a similar pattern to the outbreak incidence, declining from 4.03 (95% CI: 3.80–4.26) in 2009 to 0.24 (95% CI: 0.19–0.29) in 2022, with a peak of 13.34 (95% CI: 12.88–13.68) per million population–years in 2015. After maintaining rates of approximately 2.37–3.42 from 2016 onward, incidence rates dropped to their lowest levels during the COVID–19 pandemic years (0.96 in 2020 and 0.20 in 2021), before stabilizing at 0.24 per million population–years in 2022. Within outbreaks that included cases from both females and males, females consistently had higher incidence rates than males (Figure 2a; Supplementary Table S7).

**Figure 3:**
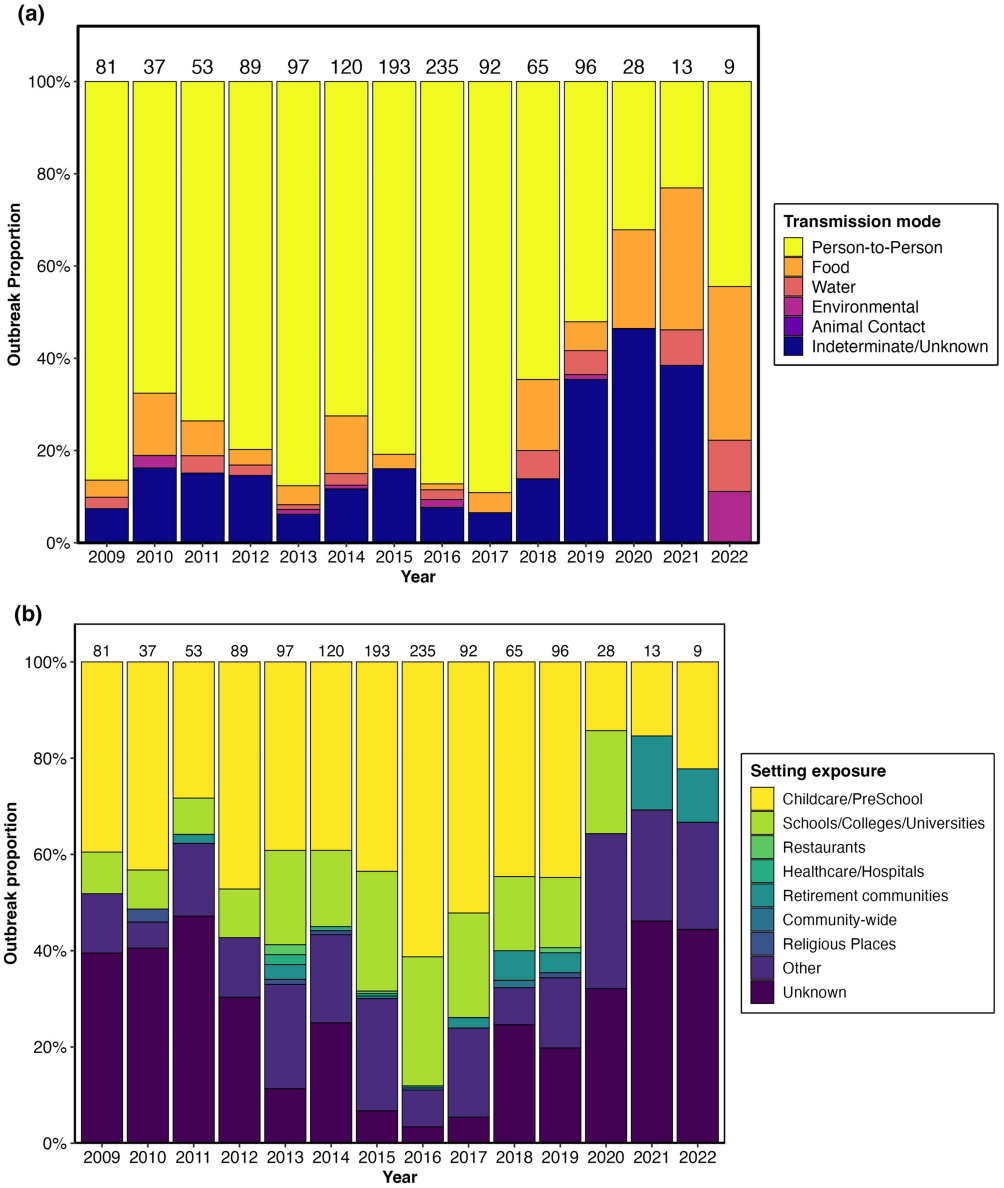
Reported *Shigella* outbreaks in the United States, 2009–2022: **(a)** Transmission mode; **(b)** exposure setting.

Among the outbreaks involving multiple age groups, the highest incidence was consistently observed in children aged < 10 years, especially in the 1–4-year age group (Figure 2b; Supplementary Table S7). Within each age group, females had a higher incidence rate than males, except for those aged 75 years and older, where the difference was minimal. Person–to-person transmission predominated throughout the study period, accounting for 52.1 to 89.1% of the outbreaks between 2009 and 2019, peaking at 89.1% in 2017 and declining to 52.1% by 2019. The person–to-person transmission rate decreased to 32.1% in 2020 and 23.1% in 2021 during the COVID-19 pandemic, before increasing to 44.4% in 2022. Indeterminate/unknown modes were the second most common mode, ranging from 6.2 to 46.4%, with a peak of 46.4% in 2020. Foodborne transmission contributed 1.3 to 33.3%, showing variability but no clear trend, whereas waterborne and environmental modes remained minor, accounting for less than 11.1% of outbreaks annually (Figure 3a; Supplementary Table S9). Childcare/preschool was the leading exposure setting category, accounting for 28.3 to 61.3% in the pre-2020 years and declining to 14.3 to 22.2% during the 2020–2022 period. Schools/colleges/universities ranked second, with rates ranging from 7.5 to 26.8%; ‘other’ settings ranked third, with rates ranging from 5.4 to 32.1%; and unknown settings ranked fourth, with rates ranging from 11.3 to 47.2%. Retirement communities, healthcare facilities/hospitals, religious institutions, restaurants, and community-wide events collectively accounted for less than 6.2% of the total (Figure 3b; Supplementary Table S10).

We identified clear seasonal patterns in *Shigella* outbreaks, with the highest outbreak counts occurring in July (119 outbreaks, 9.9%), September (121, 10.0%), and October (124, 10.3%), and the lowest in November (70, 5.8%) and December (74, 6.1%) from 2009 to 2022 (Supplementary Table S11). We identified seasonal joinpoint trends in *Shigella* outbreak frequency, revealing an increasing trend until October, with an APC of 3.03% (95% CI: 0.76 to 7.60, *p* = 0.013), and a decreasing trend thereafter, with an APC of −25.79% (95% CI: −37.31 to −9.21, *p* < 0.05) (Supplementary Figure S4). We found no clear seasonal pattern in transmission modes, with person-to-person transmission being the dominant mode across all months. We identified a significant portion of the seasonality to exposure settings, with childcare and preschool settings being implicated in 38.7 to 57.1% of the monthly outbreaks between March and August. These outbreaks peaked in April (57.1%) and June (56.8%). In contrast, we observed that educational institutions, including schools, colleges, and universities, accounted for 34.7% of the outbreaks in September and 30.0% in November. However, their contribution was less than 2% in June and July. The other settings accounted for 10.6 to 21.5% of the monthly outbreaks, whereas the unknown settings ranged from 10.9 to 31.9% of the monthly outbreaks. Settings such as retirement communities, healthcare facilities, religious places, restaurants, and community-wide events collectively accounted for an average of 4.3%.

### 3.3 Joinpoint regression trends in outbreak incidence

**Figure 4:**
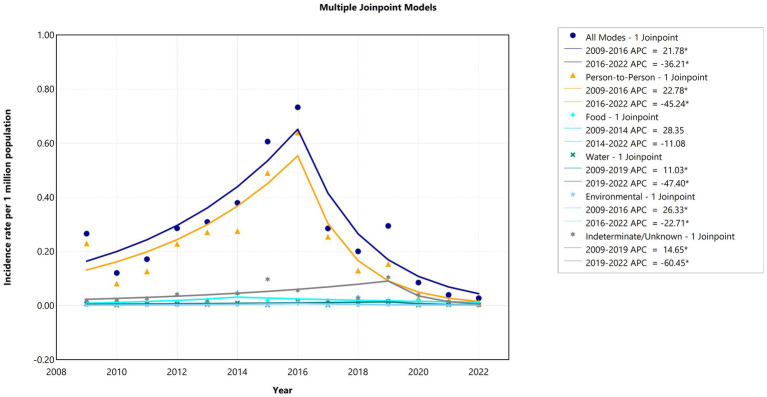
Joinpoint trends in *Shigella* outbreak incidence rates by transmission mode in the United States, 2009–2022.

Figure 4 presents the aggregate joinpoint regression analysis of *Shigella* outbreak rates per million population years, categorized by the primary mode of transmission. The overall model detected a single joinpoint in 2016, revealing a significant increase from 2009 to 2016, with an APC of 21.78% (95% CI: 11.98–42.0, *p* < 0.0002), followed by a substantial decrease from 2016 to 2022, with an APC of −36.2% (95% CI: −58.97 to −25.27, *p* < 0.001). The AAPC was −9.64% (95% CI: −19.6 to −2.7, *p* = 0.015).

Person–to–person transmission displayed a parallel joinpoint in 2016, with an APC of 22.8% (95% CI: 12.8–42.46, *p* < 0.001) in the first segment and −45.24% (95% CI: −69.4 to −33.96, *p* < 0.001) in the second segment, resulting in an AAPC of −15.4% (95% CI: −28.7 to −7.9, *p* < 0.001). The percentage of foodborne transmission APCs decreased from 28.35% between 2009 and 2014 to 17.28% between 2014 and 2022. *Shigella sonnei* exhibited the same trends as those associated with person–to–person transmission, and other *Shigella* species did not show any significant trends (Figure 4; Supplementary Figure S3; Supplementary Table S1).

### 3.4 Joinpoint regression trends in outbreak–associated case incidence

The joinpoint model demonstrated a significant (*p* < 0.05) decrease in the incidence rates, with an AAPC of −14.95% (95% CI: −28.21, −7.63) over the entire study period (2009–2022) (Figure 4). We observed two significant trends in incidence rates over time: an increase from 2009 to 2015 (APC = 22.73%; 95% CI: 10.65, 46.12) and a decrease from 2015 to 2022 (APC = −35.65%; 95% CI: −53.90, −27.10) (Supplementary Figure S2; Supplementary Table S3).

**Figure 5:**
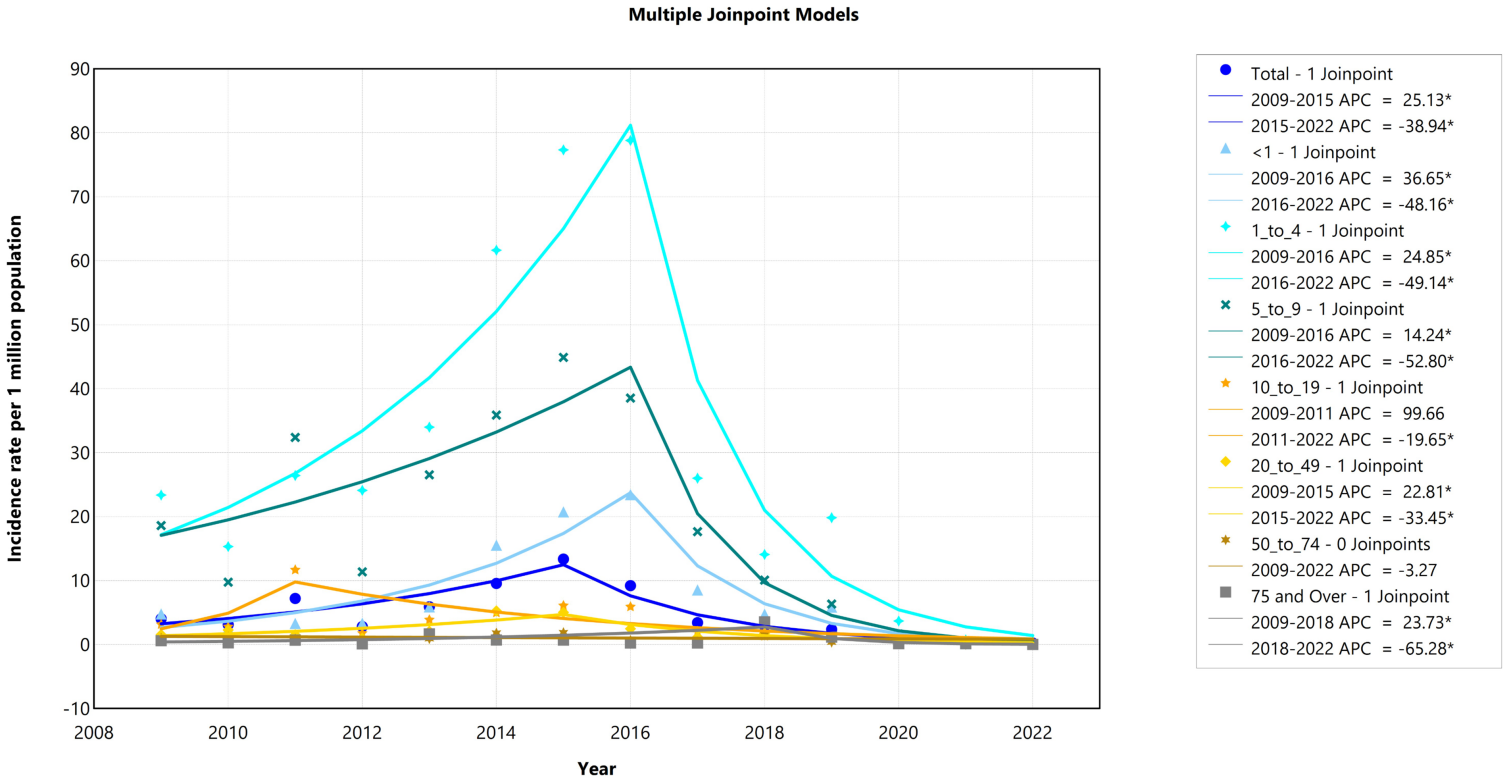
Joinpoint trends in *Shigella* outbreak–associated case incidence rates among age groups in the United States, 2009–2022.

Between 2009 and 2016, a significant increase in incidence was notable among children aged <1 year (36.6%), those aged 1–4 years (24.9%), those aged 5–9 years (14.2%), and adults aged 20–49 years (22.8%). Between 2016 and 2022, a decrease was observed in the same age groups: <1 year (APC: −48.2%), 1–4 years (APC: −49.1%), 5–9 years (APC: −52.8%), and 20–49 years (APC: −33.5%). Interestingly, the incidence among adolescents aged 10–19 years increased from 2009 to 2011 (APC: 99.6%), then decreased steadily until 2022 (APC: −19.64%), with one joinpoint detected. The AAPC during the observed study period (2009–2022) significantly decreased only among those aged <50 years. Moreover, the incidence rate in the 50 years and above age group did not significantly differ between 2009 and 2022 (Figure 5; Supplementary Table S3).

With respect to sex, both sexes had joinpoints in 2015, with APCs before and after 2015, and the overall AAPC resembled the overall trends. Although *Shigella* outbreak-associated case incidence rates were higher among females than among males, females showed a more negative AAPC (−14.67% compared with −13.35%) (Supplementary Figure S2; Supplementary Table S3).

## 4 Discussion

This study analyzed *Shigella* outbreak data from the National Outbreak Reporting System (NORS) to calculate outbreak rates and outbreak-associated case incidence rates per 1 million population-years in the United States from 2009 to 2022. We identified annual proportions and seasonal patterns in exposure and transmission modes across outbreak settings. In addition, joinpoint regression analysis characterized temporal trends in these rates and identified a significant shift in the incidence trajectory between 2015 and 2016.

*Shigella* outbreak incidence rates per 1 million population-years peaked in 2016 (0.74, 95% CI: 0.65–0.84) and reached their lowest point in 2022 (0.03, 95% CI: 0.01–0.05), with person-to-person transmission consistently dominating outbreaks, while waterborne and environmental transmission remained negligible. *Shigella* outbreak-associated case incidence rates peaked in 2015 and similarly declined to their lowest rates in 2022. The age-specific *Shigella* incidence rate was highest among children <10 years, mirroring the overall outbreak rate and its trajectory. The 1–4-year age group, in particular, had the highest rate throughout the study, consistent with the *Shigella* burden documented in previous studies (9, 29–32).

*Shigella* outbreak and case rates peaked around 2015–2016, likely reflecting the combined effects of enhanced detection and genuine changes in transmission. Several factors contributed to this rise, including an increase in outbreaks in childcare and school settings (7) and the widespread adoption of culture-independent diagnostic tests (CIDTs), which markedly increased surveillance sensitivity and identified infections previously missed by culture-based methods (13, 14, 33). Global molecular studies indicate that culture-based testing substantially underestimates the *Shigella* burden, underscoring the impact of diagnostic practices on reported incidence, particularly among young children (31, 34). During the same period, increases in multidrug-resistant *Shigella* (5, 35, 36) and outbreaks among unhoused persons (2, 8) and men who have sex with men (MSM) (2) may have further intensified transmission. This pattern aligns with a study from the United Kingdom indicating that extensively drug-resistant *S. sonnei* and continued MSM-associated transmission contributed to epidemic growth (37). However, we observed increases across all age groups, with the rise most pronounced among children under 10 years of age. This pattern suggests that intensified transmission within childcare and school settings, driven by both natural increases in spread and the heightened sensitivity of newer diagnostic methods, played a central role in the mid-2010s peak.

The post-2016 decline in *Shigella* outbreaks and incidence rates likely reflects a combination of improved prevention practices and broader reductions in reported enteric outbreaks nationwide (6, 7). Enhanced hygiene and sanitation measures in childcare facilities may have contributed to the initial pre-pandemic decrease, while contemporaneous declines in outbreak reporting may also have influenced national trends. The pronounced reduction observed during 2020–2022 aligns with the effects of COVID-19 mitigation measures, including social distancing, enhanced hygiene, and childcare and school closures, all of which suppressed fecal–oral pathogen transmission (17, 38). The sharp decrease in foodborne and person-to-person enteric infections during the pandemic period may additionally reflect changes in health-seeking behavior, reduced dining out, disruptions in diagnostic testing, and diminished capacity for outbreak surveillance and reporting (39, 40). Accordingly, trends observed during the pandemic years should be interpreted with caution, as this exceptional period may not fully represent outbreak dynamics under usual surveillance and transmission conditions.

Underlying medical conditions and concurrent infections may further shape susceptibility to shigellosis and affect clinical severity, particularly among immunocompromised or otherwise medically vulnerable populations (41–43). Evidence specific to *Shigella* spp. indicates that invasive outcomes such as bacteremia are uncommon but occur more frequently in higher-risk groups, including persons with HIV and individuals with comorbidities such as diabetes, cirrhosis, malignancy, and other immunosuppressive conditions (44, 45). HIV infection, in particular, has been identified as a factor that may increase both acquisition and transmission risk through prolonged carriage or altered immune responses (46). Long COVID has been increasingly linked to persistent immune dysfunction, chronic inflammation, and autoimmune mechanisms, which may potentially alter the risk of subsequent infections, including enteric bacterial diseases such as shigellosis (47, 48). Epidemiologic cohort studies have similarly assessed the post-acute burden of infections and other health outcomes following COVID-19, underscoring the broader infectious disease implications of pandemic-era immune perturbations (41). However, NORS does not capture prior SARS-CoV-2 infection history or long COVID status; therefore, these mechanisms cannot be directly assessed within the present outbreak-based analysis.

Although NORS does not systematically collect patient-level data on comorbidities or co-infections, co-circulating enteric pathogens and microbial interactions may also contribute to variation in susceptibility and clinical presentation. Prior studies have reported associations between viral pathogens, such as rotavirus and *Shigella* spp., suggesting that viral–bacterial interactions may influence diarrheal disease dynamics (49). Taken together, these host- and pathogen-related factors may contribute to differences in outbreak impact and warrant further investigation using linked clinical and laboratory surveillance data.

Our analysis of *Shigella* outbreaks revealed a clear seasonal trend, with 9.3–10.3% of annual outbreaks occurring from May to October, peaking in summer and fall due to increased social interactions in childcare and school/college/university settings, consistent with prior studies (6, 12, 50, 51). Childcare and preschool settings reported outbreaks year-round, whereas school, college, and university settings showed higher numbers in the fall months and markedly lower numbers in the summer, reflecting the dynamics of the academic calendar and periods of intensified contact among susceptible populations.

These findings are subject to several limitations. First, reporting *Shigella* outbreaks to NORS is voluntary, and not all variables are required, which may result in underreporting and incomplete information for key fields (e.g., age and sex), potentially underestimating their contribution to outbreak patterns. The adoption of CIDTs during the 2015–2016 period likely increased detection sensitivity, potentially biasing comparisons of trends before and after this transition. In later years, as CIDT use became more standardized, detection rates likely remained higher than pre-2015 levels, potentially making earlier years appear artificially lower. Although the post-peak decline aligns with broader reductions in enteric infections, these diagnostic shifts complicate the interpretation of long-term trends. NORS data are also affected by differences in surveillance methods, diagnostic capacity, and investigative resources across states. Additionally, NORS captures only outbreak-associated cases and omits sporadic shigellosis, which accounts for the majority of US infections and may follow different epidemiologic patterns, particularly among populations such as unhoused individuals or specific sexual networks, who may be underrepresented in outbreak investigations. Future work could integrate NORS data with other surveillance systems, including laboratory-based case reporting and antimicrobial resistance monitoring, to more fully characterize national transmission patterns and the contribution of emerging drug-resistant lineages.

These findings extend prior US descriptions of shigellosis by quantifying temporal changes and linking them to demographic and environmental factors (6, 7, 52). The dominance of person–to–person transmission and childcare–associated outbreaks underscores the need for targeted interventions. Behavioral and institutional factors strongly influence the success of these interventions, as sustained improvements in hand hygiene and environmental cleaning depend on consistent reinforcement, training, and administrative support. Evidence from broader public health research shows that preventive behaviors are most effective when individuals are supported by clear policies, adequate resources, and routine educational reminders (53, 54). Based on these findings, we recommend implementing year-round *Shigella* prevention education in childcare facilities, focusing on handwashing protocols and staff training, as well as issuing reminders to schools and universities before the start of the fall semester to reinforce hygiene practices. Additional efforts to address multidrug-resistant *Shigella* and to support prevention in high-risk groups such as unhoused individuals and men who have sex with men (MSM) remain essential.

## Data Availability

Publicly available datasets were analyzed in this study. This data can be found here: https://www.cdc.gov/ncezid/dfwed/BEAM-dashboard.html CDC NORS.
