# Supplementary material for: Temporal trends of Shigella outbreaks in the United States, 2009–2022
Source: Front Public Health. 2026 Feb 12;14:1740303. doi: 10.3389/fpubh.2026.1740303 (PMC12936016; doi:10.3389/fpubh.2026.1740303)
Supplement: Supplementary file 1 [file Table_1.DOCX]

**Temporal Trends of *Shigella* Outbreaks in the United States, 2009–2022**

**Supplementary information**

Table S1. Join-point regression model of *Shigella* outbreak incidence rates by transmission mode in the US, 2009–2022.

| Cohort | Time period | APC, 95% CI | AAPC, 95% CI | *p* value |
| --- | --- | --- | --- | --- |
| Total | 2009­­–2016 | 21.78, 11.98 – 42.00 |  | < 0.001 |
|  | 2016–2022 | -36.21, -58.97 – -25.27 |  | < 0.001 |
|  | 2009–2022 |  | -9.64, -19.66 – -2.72 | 0.015 |
| Transmission mode |  |  |  |  |
| Person-to-person | 2009–2016 | 22.77, 12.78 – 42.46 |  | < 0.001 |
|  | 2016–2022 | -45.24, -69.47 – -33.96 |  | < 0.001 |
|  | 2009–2022 |  | -15.42, -28.83 – -7.79 | 0.001 |
| Food | 2009–2014 | 28.35, -0.23 – 494.32 |  | 0.052 |
|  | 2014–2022 | -11.07, -76.51 – 1.28 |  | 0.06 |
|  | 2009–2022 |  | 2.40, -17.79 – 24.07 | 0.658 |
| Water | 2009–2019 | 11.02, 3.33 – 44.59 |  | 0.014 |
|  | 2009–2022 | -47.40, -86.93 – -12.42 |  | 0.014 |
|  | 2009–2022 |  | -6.55, -20.91 – 6.77 | 0.244 |
| Environmental | 2009–2016 | 26.32, 10.03 – 154.29 |  | 0.004 |
|  | 2016–2022 | -22.70, -59.35 – -7.27 |  | 0.005 |
|  | 2009–2022 |  | 0.69, -10.68 – 15.93 | 0.859 |
| Indeterminate/Unknown | 2009–2019 | 14.64, 5.21 – 46.27 |  | 0.01 |
|  | 2019–2022 | -60.45, -94.94 – -8.28 |  | 0.022 |
|  | 2009–2022 |  | -10.32, -30.28 – 9.70 | 0.179 |

Table S2. Join-point regression model of *Shigella* outbreak incidence rates by species in the US, 2009–2022.

| Cohort | Time period | APC, 95% CI | AAPC, 95% CI | *p* value |
| --- | --- | --- | --- | --- |
| *Shigella* species |  |  |  |  |
| *Shigella sonnei* | 2009–2016 | 19.77, 10.51 – 38.46 |  | < 0.001 |
|  | 2016–2022 | -42.56, -67.17 – -31.86 |  | < 0.001 |
|  | 2009–2022 |  | -14.68, -27.24 – -8.05 | 0.002 |
| *Shigella flexneri* | 2009–2022 | -0.64, -5.94 – 4.95 |  | 0.829 |
|  | 2009–2022 |  | -0.64, -5.94 – 4.95 | 0.829 |
| *Shigella boydii* | 2009–2022 | -2.02, -5.58 – 1.57 |  | 0.23 |
|  | 2009–2022 |  | -2.02, -5.58 – 1.57 | 0.23 |
| *Shigella dysenteriae* | 2009–2022 | -0.93, -4.68 – 2.82 |  | 0.527 |
|  | 2009–2022 |  | -0.93, -4.68 – 2.82 | 0.527 |
| *Shigella* multi-species | 2009–2018 | 24.44, 17.59 – 41.45 |  | < 0.001 |
|  | 2018–2022 | -37.35, -59.12 – -24.69 |  | < 0.001 |
|  | 2009–2022 |  | 0.75, -7.85 – 8.51 | 0.702 |
| *Shigella* unknown | 2009–2019 | 23.10, 12.83 – 821.05 |  | 0.002 |
|  | 2019–2022 | -62.33, -97.12 – -3.62 |  | 0.037 |
|  | 2009–2022 |  | -6.33, -29.76 – 82.79 | 0.565 |

Table S3. Join-point regression model of *Shigella* outbreak-associated case incidence rates in the US, 2009–2022.

| Cohort | Time period | APC, 95% CI | AAPC, 95% CI | *p* value |
| --- | --- | --- | --- | --- |
| Total | 2009–2015 | 25.12, 11.47 – 53.76 |  | 0.001 |
|  | 2015–2022 | -38.91, -60.07 – -29.57 |  | < 0.001 |
|  | 2009–2022 |  | -14.95, -28.21 – -7.63 | 0.004 |
| Sex |  |  |  |  |
| Male | 2009–2015 | 22.58, 10.65 – 46.12 |  | 0.001 |
|  | 2015–2022 | -35.65, -53.90 – -27.10 |  | < 0.001 |
|  | 2009–2022 |  | -13.35, -24.33 – -7.22 | 0.001 |
| Female | 2009–2015 | 22.73, 9.25 – 50.64 |  | 0.002 |
|  | 2015–2022 | -37.51, -60.83 – -27.61 |  | < 0.001 |
|  | 2009–2022 |  | -14.67, -28.70 – -7.61 | 0.004 |
| Age group (Year) |  |  |  |  |
| <1 | 2009–2016 | 36.64, 23.67 – 68.59 |  | < 0.001 |
|  | 2016–2022 | -48.16, -78.18 – -33.83 |  | < 0.001 |
|  | 2009–2022 |  | -12.63, -33.35 – -1.56 | 0.032 |
| 1–4 | 2009–2016 | 24.85, 15.41 – 42.22 |  | < 0.001 |
|  | 2016–2022 | -49.13, -73.07 – -36.77 |  | < 0.001 |
|  | 2009–2022 |  | -17.51, -32.20 – -9.70 | 0.002 |
| 5–9 | 2009–2016 | 14.24, 4.74 – 28.89 |  | 0.004 |
|  | 2016–2022 | -52.80, -77.06 – -38.30 |  | < 0.001 |
|  | 2009–2022 |  | -24.02, -39.71 – -15.64 | < 0.001 |
| 10–19 | 2009–2011 | 99.66, -11.78 – 359.57 |  | 0.191 |
|  | 2011–2022 | -19.64, -98.65 – -14.07 |  | 0.002 |
|  | 2009–2022 |  | -7.56, -57.43 – 2.35 | 0.098 |
| 20–49 | 2009–2015 | 22.80, 5.75 – 80.05 |  | 0.021 |
|  | 2015–2022 | -33.45, -72.43 – -21.46 |  | < 0.001 |
|  | 2009–2022 |  | -11.70, -33.98 – -0.85 | 0.043 |
| 50–74 | 2009–2022 | -3.26, -15.23 – 8.98 |  | 0.494 |
|  | 2009–2022 |  | -3.26, -15.23 – 8.98 | 0.494 |
| ≥75 | 2009–2018 | 23.72, 7.86 – 108.29 |  | 0.015 |
|  | 2018–2022 | -65.28, -99.87 – -18.83 |  | 0.028 |
|  | 2009–2022 |  | -16.31, -60.73 – 9.51 | 0.494 |

Table S4. Join-point regression model of *Shigella* outbreak seasonality in the US, 2009–2022.

| Cohort | Time period | APC, 95% CI | AAPC, 95% CI | *p* value |
| --- | --- | --- | --- | --- |
|  |  |  |  |  |
| Outbreaks | January–October | 3.02, 0.76 – 7.60 |  | 0.013 |
|  | October–December | -25.79, -37.31 – -9.21 |  | < 0.001 |
|  | January–December |  | -2.93, -5.55 – -0.25 | 0.035 |
| Transmission mode |  |  |  |  |
| Person-to-person | January–September | 2.67, 0.63 – 5 |  | 0.011 |
|  | September–December | -20.91, -35.68 – -13.49 |  | < 0.001 |
|  | January–December |  | -4.38, -6.71 – -2.94 | < 0.001 |
| Food | January–April | -16.18, -66.08 – 46.33 |  | 0.293 |
|  | April–July | 109.18, 20.57 – 206.08 |  | 0.034 |
|  | July–December | -14.60, -60.28 – 7.43 |  | 0.122 |
|  | January–December |  | 8.48, -5.89 – 21.01 | 0.306 |
| Water | January–June | 78.46, 30.13 – 210.49 |  | < 0.001 |
|  | June–December | -40.70, -61.37 – -24.80 |  | < 0.001 |
|  | January–December |  | -2.15, -15.06 – 12.02 | 0.639 |
| Environmental | January–December | 4.06, -5.68 – 13.38 |  | 0.408 |
|  | January–December |  | 4.06, -5.68 – 13.38 | 0.408 |
| Indeterminate/Unknown | January–December | 3.83, -0.09 – 7.62 |  | 0.054 |
|  | January–December |  | 3.83, -0.09 – 7.62 | 0.054 |

Table S5. Join-point regression model of *Shigella* outbreaks by exposure settings in the US, 2009–2022.

| Cohort | Time period | APC, 95% CI | AAPC, 95% CI | *p* value |
| --- | --- | --- | --- | --- |
| Exposure settings |  |  |  |  |
| Childcare/Preschool | 2009–2016 | 34.96, 14.37 – 70.23 |  | 0.001 |
|  | 2016–2022 | -51.12, -64.58 – -39.66 |  | < 0.001 |
|  | 2009–2022 |  | -15.54, -22.59 – -8.19 | 0.001 |
| Schools/Colleges/Universities | 2009–2016 | 51.05, 25.06 – 94.16 |  | < 0.001 |
|  | 2016–2022 | -53.97, -66.82 – -41.41 |  | < 0.001 |
|  | 2009–2022 |  | -12.71, -20.22 – -4.22 | 0.007 |
| Healthcare/Hospitals | 2009–2022 | 0.51, -5.35 – 6.66 |  | 0.852 |
|  | 2009–2022 |  | 0.51, -5.35 – 6.66 | 0.852 |
| Restaurants | 2009–2022 | -0.09, -14.74 – 16.27 |  | 0.987 |
|  | 2009–2022 |  | -0.09, -14.74 – 16.27 | 0.987 |
| Retirement communities | 2009–2018 | 20.69, 11.33 – 75.61 |  | 0.006 |
|  | 2018–2022 | -20.47, -66.02 – 4.39 |  | 0.13 |
|  | 2009–2022 |  | 6.16, -3.92 – 16.32 | 0.235 |
| Community-wide | 2009–2012 | -1.00, -15.14 – 9.45 |  | 0.669 |
|  | 2012–2016 | 19.36, -0.47 – 32.55 |  | 0.051 |
|  | 2016–2022 | -10.44, -17.89 – -5.54 |  | 0.02 |
|  | 2009–2022 |  | 0.12, -2.10 – 1.82 | 0.881 |
| Religious Places | 2009–2022 | -1.17, -9.09 – 7.34 |  | 0.757 |
|  | 2009–2022 |  | -1.17, -9.09 – 7.34 | 0.757 |
| Other | 2009–2015 | 50.11, 29.54 – 86.86 |  | < 0.001 |
|  | 2015–2022 | -29.48, -40.23 – -20.48 |  | < 0.001 |
|  | 2009–2022 |  | -0.06, -5.96 – 6.35 | 0.944 |
| Unknown | 2009–2016 | -1.53, -5.01 – 2.11 |  | 0.4 |
|  | 2016–2019 | 141.72, 121.06 – 162.55 |  | < 0.001 |
|  | 2019–2022 | -52.82, -58.13 – -47.42 |  | < 0.001 |
|  | 2009–2022 |  | 2.22, -0.12 – 4.31 | 0.063 |

Table S6. *Shigella* outbreak incidence rate per 1 million population years by transmission mode in the US, 2009–2022.

|  | Outbreak incidence rate, 95% CI | | | | | |  |
| --- | --- | --- | --- | --- | --- | --- | --- |
| Year | Total | Person – to – Person | Food | Water | Environmental | Indeterminate/Unknown | |
| 2009 | 0.27, 0.21 – 0.33 | 0.23, 0.18 – 0.29 | 0.01, 0 – 0.03 | 0.01, 0 – 0.02 | 0, 0 – 0.01 | 0.02, 0.01 – 0.04 | |
| 2010 | 0.12, 0.08 – 0.17 | 0.08, 0.05 – 0.12 | 0.02, 0.01 – 0.04 | 0, 0 – 0.01 | 0, 0 – 0.02 | 0.02, 0.01 – 0.04 | |
| 2011 | 0.17, 0.13 – 0.22 | 0.13, 0.09 – 0.17 | 0.01, 0 – 0.03 | 0.01, 0 – 0.02 | 0, 0 – 0.01 | 0.03, 0.01 – 0.05 | |
| 2012 | 0.29, 0.23 – 0.35 | 0.23, 0.18 – 0.29 | 0.01, 0 – 0.03 | 0.01, 0 – 0.02 | 0, 0 – 0.01 | 0.04, 0.02 – 0.07 | |
| 2013 | 0.31, 0.25 – 0.38 | 0.27, 0.22 – 0.33 | 0.01, 0 – 0.03 | 0, 0 – 0.02 | 0, 0 – 0.02 | 0.02, 0.01 – 0.04 | |
| 2014 | 0.38, 0.31 – 0.45 | 0.28, 0.22 – 0.34 | 0.05, 0.03 – 0.08 | 0.01, 0 – 0.03 | 0, 0 – 0.02 | 0.04, 0.02 – 0.07 | |
| 2015 | 0.61, 0.52 – 0.7 | 0.49, 0.42 – 0.57 | 0.02, 0.01 – 0.04 | 0, 0 – 0.01 | 0, 0 – 0.01 | 0.1, 0.07 – 0.14 | |
| 2016 | 0.73, 0.64 – 0.83 | 0.64, 0.55 – 0.73 | 0.01, 0 – 0.03 | 0.02, 0.01 – 0.04 | 0.01, 0 – 0.03 | 0.06, 0.03 – 0.09 | |
| 2017 | 0.28, 0.23 – 0.35 | 0.25, 0.2 – 0.32 | 0.01, 0 – 0.03 | 0, 0 – 0.01 | 0, 0 – 0.01 | 0.02, 0.01 – 0.04 | |
| 2018 | 0.2, 0.15 – 0.26 | 0.13, 0.09 – 0.17 | 0.03, 0.01 – 0.06 | 0.01, 0 – 0.03 | 0, 0 – 0.01 | 0.03(0.01 – 0.05 | |
| 2019 | 0.29, 0.24 – 0.36 | 0.15, 0.11 – 0.2 | 0.02, 0.01 – 0.04 | 0.02, 0 – 0.04 | 0, 0 – 0.02 | 0.1, 0.07 – 0.15 | |
| 2020 | 0.09, 0.06 – 0.12 | 0.03, 0.01 – 0.05 | 0.02, 0.01 – 0.04 | 0, 0 – 0.01 | 0, 0 – 0.01 | 0.04, 0.02 – 0.07 | |
| 2021 | 0.04, 0.02 – 0.07 | 0.01, 0 – 0.03 | 0.01, 0 – 0.03 | 0, 0 – 0.02 | 0, 0 – 0.01 | 0.02, 0 – 0.04 | |
| 2022 | 0.03, 0.01 – 0.05 | 0.01, 0 – 0.03 | 0.01, 0 – 0.03 | 0, 0 – 0.02 | 0, 0 – 0.02 | 0, 0 – 0.01 | |

Table S7. *Shigella* outbreak-associated case incidence rate per 1 million population in the US, 2009–2022.

|  | Incidence rate, 95% CI | | |  |
| --- | --- | --- | --- | --- |
| Year | Total | Male | Female | |
| 2009 | 4.03, 3.8 – 4.26 | 3.47, 3.18 – 3.78 | 3.69, 3.4 – 4.01 | |
| 2010 | 3.06, 2.87 – 3.26 | 2.97, 2.7 – 3.25 | 3.13, 2.86 – 3.42 | |
| 2011 | 7.19, 6.9 – 7.5 | 5.36, 5 – 5.74 | 6.1, 5.72 – 6.5 | |
| 2012 | 2.81, 2.62 – 3 | 2.78, 2.52 – 3.06 | 2.66, 2.41 – 2.92 | |
| 2013 | 5.86, 5.59 – 6.13 | 5.27, 4.91 – 5.65 | 6.19, 5.82 – 6.59 | |
| 2014 | 9.55, 9.21 – 9.9 | 7.83, 7.4 – 8.29 | 9.28, 8.82 – 9.76 | |
| 2015 | 13.3, 12.91 – 13.71 | 10.07, 9.57 – 10.57 | 10.31, 9.82 – 10.82 | |
| 2016 | 9.2, 8.87 – 9.53 | 7.23, 6.82 – 7.66 | 7.9, 7.47 – 8.34 | |
| 2017 | 3.42, 3.22 – 3.63 | 2.88, 2.62 – 3.16 | 2.82, 2.57 – 3.09 | |
| 2018 | 2.7, 2.52 – 2.88 | 2.45, 2.21 – 2.71 | 2.86, 2.61 – 3.13 | |
| 2019 | 2.37, 2.21 – 2.54 | 2.03, 1.81 – 2.26 | 2.28, 2.06 – 2.52 | |
| 2020 | 0.96, 0.86 – 1.07 | 1.28, 1.11 – 1.46 | 0.61, 0.49 – 0.74 | |
| 2021 | 0.2, 0.15 – 0.25 | 0.15, 0.1 – 0.23 | 0.21, 0.15 – 0.29 | |
| 2022 | 0.24, 0.19 – 0.29 | 0.19, 0.13 – 0.27 | 0.25, 0.18 – 0.34 | |

Table S8. *Shigella* Outbreak-associated case incidence rate per 1 million population by age group (years) in the US, 2009–2022.

|  | Incidence rate, 95% CI | | | | | | |  |
| --- | --- | --- | --- | --- | --- | --- | --- | --- |
| Year | <1 | 1 – 4 | 5 – 9 | 10 – 19 | 20 – 49 | 50 – 74 | ≥75 | |
| 2009 | 4.76, 2.87 – 7.43 | 23.27, 20.98 – 25.75 | 18.53, 16.69 – 20.51 | 3.13, 2.63 – 3.71 | 1.55, 1.34 – 1.79 | 0.97, 0.76 – 1.21 | 0.6, 0.3 – 1.07 | |
| 2010 | 2.54, 1.22 – 4.67 | 14.7, 12.89 – 16.69 | 9.57, 8.27 – 11.01 | 2.78, 2.3 – 3.33 | 2.25, 2 – 2.53 | 1.15, 0.93 – 1.41 | 0.27(0.09 – 0.63 | |
| 2011 | 3.29, 1.75 – 5.63 | 26.31, 23.86 – 28.94 | 32.25, 29.82 – 34.82 | 11.63, 10.63 – 12.71 | 1.52, 1.32 – 1.76 | 0.86, 0.67 – 1.08 | 0.69, 0.37 – 1.18 | |
| 2012 | 3.32, 1.77 – 5.68 | 23.99, 21.65 – 26.52 | 11.32, 9.9 – 12.87 | 1.77, 1.39 – 2.22 | 1, 0.83 – 1.19 | 0.29, 0.19 – 0.43 | 0.1, 0.01 – 0.38 | |
| 2013 | 5.87, 3.72 – 8.80 | 33.39, 30.61 – 36.36 | 26.27, 24.1 – 28.59 | 3.79, 3.22 – 4.43 | 2.04, 1.8 – 2.31 | 0.92, 0.73 – 1.15 | 1.65(1.13 – 2.33 | |
| 2014 | 15.47, 11.83 – 19.87 | 61.36, 57.57 – 65.34 | 35.72, 33.18 – 38.41 | 4.96, 4.3 – 5.68 | 5.26, 4.86 – 5.67 | 1.78, 1.52 – 2.08 | 0.71, 0.39 – 1.19 | |
| 2015 | 20.64, 16.42 – 25.62 | 76.78, 72.53 – 81.21 | 44.6, 41.75 – 47.6 | 6.05, 5.32 – 6.84 | 4.98, 4.6 – 5.38 | 1.84, 1.57 – 2.14 | 0.7, 0.38 – 1.17 | |
| 2016 | 23.33, 18.81 – 28.61 | 78.25, 73.97 – 82.72 | 38.32, 35.68 – 41.1 | 5.89, 5.18 – 6.68 | 2.46, 2.19 – 2.74 | 0.64, 0.49 – 0.83 | 0.24, 0.08 – 0.57 | |
| 2017 | 8.50, 5.85 – 11.93 | 25.89, 23.45 – 28.51 | 17.57, 15.8 – 19.5 | 2.06, 1.65 – 2.55 | 1.18, 1 – 1.39 | 0.39, 0.27 – 0.54 | 0.24, 0.08 – 0.55 | |
| 2018 | 4.47, 2.60 – 7.15 | 13.52, 11.77 – 15.45 | 9.68, 8.37 – 11.14 | 1.29, 0.97 – 1.69 | 1.65, 1.43 – 1.89 | 1.13, 0.92 – 1.36 | 3.43, 2.69 – 4.29 | |
| 2019 | 5.83, 3.65 – 8.83 | 19.74, 17.61 – 22.06 | 6.3, 5.26 – 7.5 | 1.08, 0.79 – 1.45 | 0.77, 0.63 – 0.94 | 0.38, 0.27 – 0.53 | 1.15(0.75 – 1.69 | |
| 2020 | 0.27, 0.01 – 1.51 | 3.67, 2.78 – 4.75 | 1.63, 1.12 – 2.28 | 0.44, 0.27 – 0.69 | 1, 0.83 – 1.19 | 0.71, 0.55 – 0.9 | 0.14, 0.03 – 0.4 | |
| 2021 | 0, 0 – 1.04 | 0.72, 0.36 – 1.29 | 0.59, 0.31 – 1.03 | 0.14, 0.05 – 0.3 | 0.22, 0.15 – 0.32 | 0.04, 0.01 – 0.11 | 0.14, 0.03 – 0.39 | |
| 2022 | 0.27, 0.01 – 1.52 | 0.47, 0.19 – 0.96 | 0.15, 0.03 – 0.43 | 0.05, 0.01 – 0.17 | 0.03, 0.01 – 0.08 | 0.07, 0.03 – 0.15 | 0, 0 – 0.16 | |

Table S9. *Shigella* outbreaks by transmission mode in the US, 2009–2022.

|  | Outbreak no. (%) | | | | |
| --- | --- | --- | --- | --- | --- |
| Year | Person to person | Food | Water | Environmental | Indeterminate/Unknown |
| 2009 | 70 (86.4) | 3 (3.7) | 2 (2.5) | 0 (0) | 6 (7.4) |
| 2010 | 25 (67.6) | 5 (13.5) | 0 (0) | 1 (2.7) | 6 (16.2) |
| 2011 | 39 (73.6) | 4 (7.5) | 2 (3.8) | 0 (0) | 8 (15.1) |
| 2012 | 71 (79.8) | 3 (3.4) | 2 (2.2) | 0 (0) | 13 (14.6) |
| 2013 | 85 (87.6) | 4 (4.1) | 1 (1) | 1 (1) | 6 (6.2) |
| 2014 | 87 (72.5) | 15 (12.5) | 3 (2.5) | 1 (0.8) | 14 (11.7) |
| 2015 | 156 (80.8) | 6 (3.1) | 0 (0) | 0 (0) | 31 (16.1) |
| 2016 | 205 (87.2) | 3 (1.3) | 5 (2.1) | 4 (1.7) | 18 (7.7) |
| 2017 | 82 (89.1) | 4 (4.3) | 0 (0) | 0 (0) | 6 (6.5) |
| 2018 | 42 (64.6) | 10 (15.4) | 4 (6.2) | 0 (0) | 9 (13.8) |
| 2019 | 50 (52.1) | 6 (6.2) | 5 (5.2) | 1 (1) | 34 (35.4) |
| 2020 | 9 (32.1) | 6 (21.4) | 0 (0) | 0 (0) | 13 (46.4) |
| 2021 | 3 (23.1) | 4 (30.8) | 1 (7.7) | 0 (0) | 5 (38.5) |
| 2022 | 4 (44.4) | 3 (33.3) | 1 (11.1) | 1 (11.1) | 0 (0) |

Table S10. *Shigella* outbreaks by setting of exposure in the US, 2009–2022.

|  | Outbreak no. (%) | | | | | | | | |
| --- | --- | --- | --- | --- | --- | --- | --- | --- | --- |
| Year | Childcare/  Preschool | Schools/  Colleges/  Universities | Healthcare/  Hospitals | Restaurants | Religious  Places | Retirement  communities | Community –  wide | Other | Unknown |
| 2009 | 32 (39.5) | 7 (8.6) | 0 (0) | 0 (0) | 0 (0) | 0 (0) | 0 (0) | 10 (12.3) | 32 (39.5) |
| 2010 | 16 (43.2) | 3 (8.1) | 0 (0) | 0 (0) | 1 (2.7) | 0 (0) | 0 (0) | 2 (5.4) | 15 (40.5) |
| 2011 | 15 (28.3) | 4 (7.5) | 0 (0) | 0 (0) | 0 (0) | 1 (1.9) | 0 (0) | 8 (15.1) | 25 (47.2) |
| 2012 | 42 (47.2) | 9 (10.1) | 0 (0) | 0 (0) | 0 (0) | 0 (0) | 0 (0) | 11 (12.4) | 27 (30.3) |
| 2013 | 38 (39.2) | 19 (19.6) | 2 (2.1) | 2 (2.1) | 1 (1) | 3 (3.1) | 0 (0) | 21 (21.6) | 11 (11.3) |
| 2014 | 47 (39.2) | 19 (15.8) | 1 (0.8) | 0 (0) | 1 (0.8) | 0 (0) | 0 (0) | 22 (18.3) | 30 (25) |
| 2015 | 84 (43.5) | 48 (24.9) | 1 (0.5) | 1 (0.5) | 0 (0) | 1 (0.5) | 0 (0) | 45 (23.3) | 13 (6.7) |
| 2016 | 144 (61.3) | 63 (26.8) | 0 (0) | 0 (0) | 0 (0) | 1 (0.4) | 1 (0.4) | 18 (7.7) | 8 (3.4) |
| 2017 | 48 (52.2) | 20 (21.7) | 0 (0) | 0 (0) | 0 (0) | 2 (2.2) | 0 (0) | 17 (18.5) | 5 (5.4) |
| 2018 | 29 (44.6) | 10 (15.4) | 0 (0) | 0 (0) | 0 (0) | 4 (6.2) | 1 (1.5) | 5 (7.7) | 16 (24.6) |
| 2019 | 43 (44.8) | 14 (14.6) | 0 (0) | 1 (1) | 1 (1) | 4 (4.2) | 0 (0) | 14 (14.6) | 19 (19.8) |
| 2020 | 4 (14.3) | 6 (21.4) | 0 (0) | 0 (0) | 0 (0) | 0 (0) | 0 (0) | 9 (32.1) | 9 (32.1) |
| 2021 | 2 (15.4) | 0 (0) | 0 (0) | 0 (0) | 0 (0) | 2 (15.4) | 0 (0) | 3 (23.1) | 6 (46.2) |
| 2022 | 2 (22.2) | 0 (0) | 0 (0) | 0 (0) | 0 (0) | 1 (11.1) | 0 (0) | 2 (22.2) | 4 (44.4) |

Table S11. *Shigella* outbreaks by month in the US, 2009–2022.

| Month | Outbreak no. (%) |
| --- | --- |
| January | 91 (7.53) |
| February | 94 (7.78) |
| March | 93 (7.70) |
| April | 91 (7.53) |
| May | 114 (9.44) |
| June | 111 (9.19) |
| July | 119 (9.85) |
| August | 106 (8.77) |
| September | 121 (10.02) |
| October | 124 (10.26) |
| November | 70 (5.79) |
| December | 74 (6.13) |
| Total | 1208 (100) |


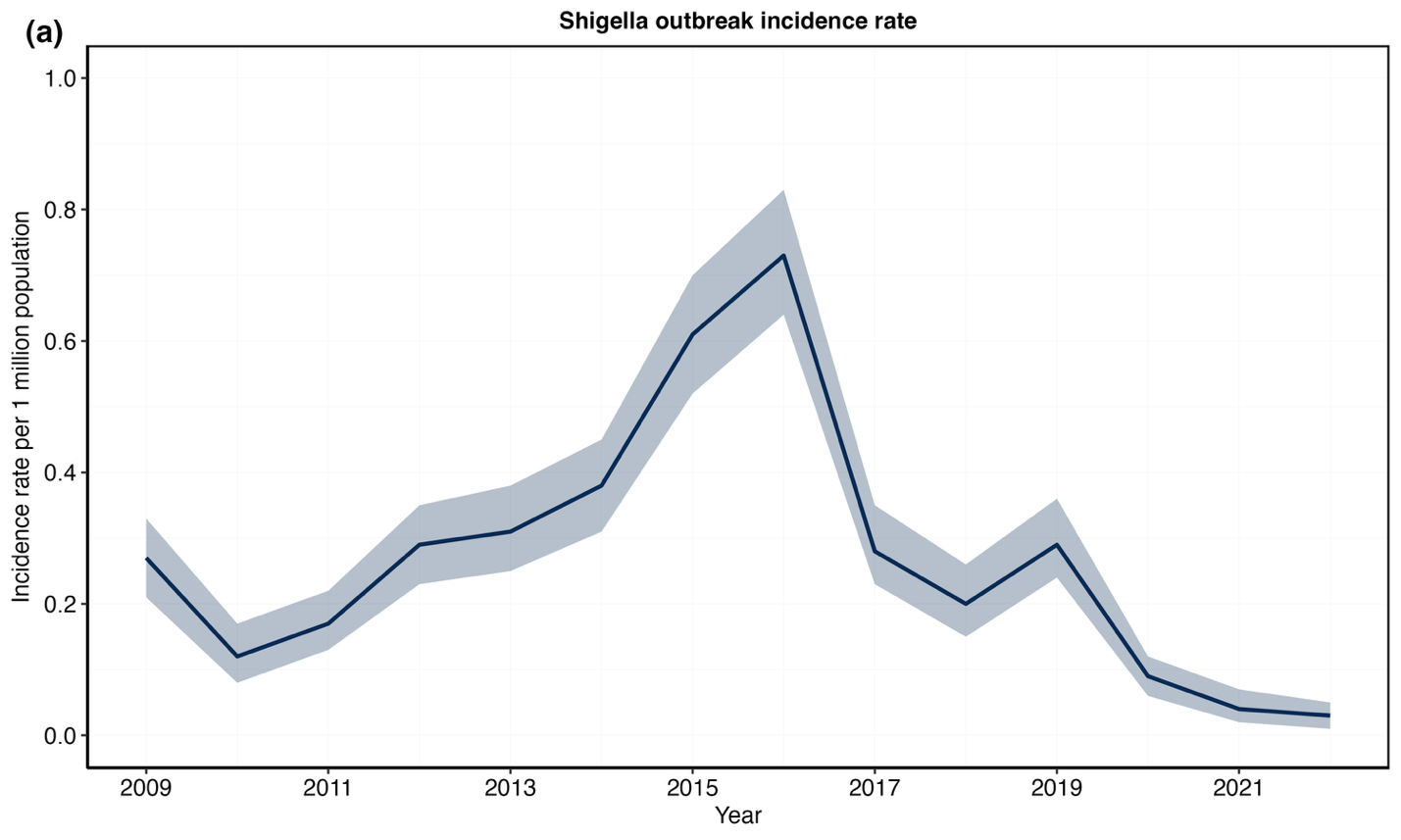

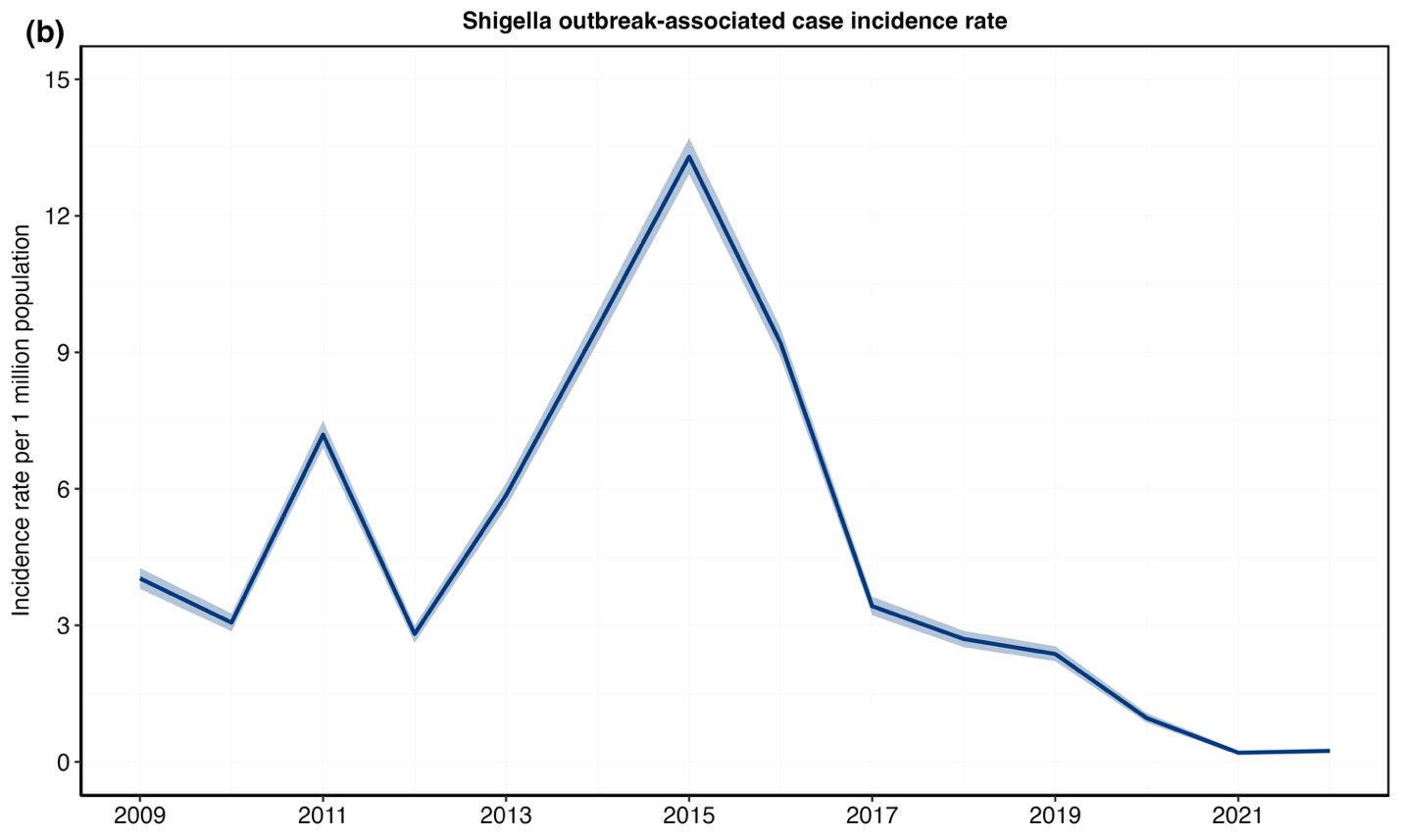


Figure S1. *Shigella* incidence rate per 1 million population in the United States, 2009–2022. (a) Outbreak incidence rate (b) Outbreak-associated case incidence rate


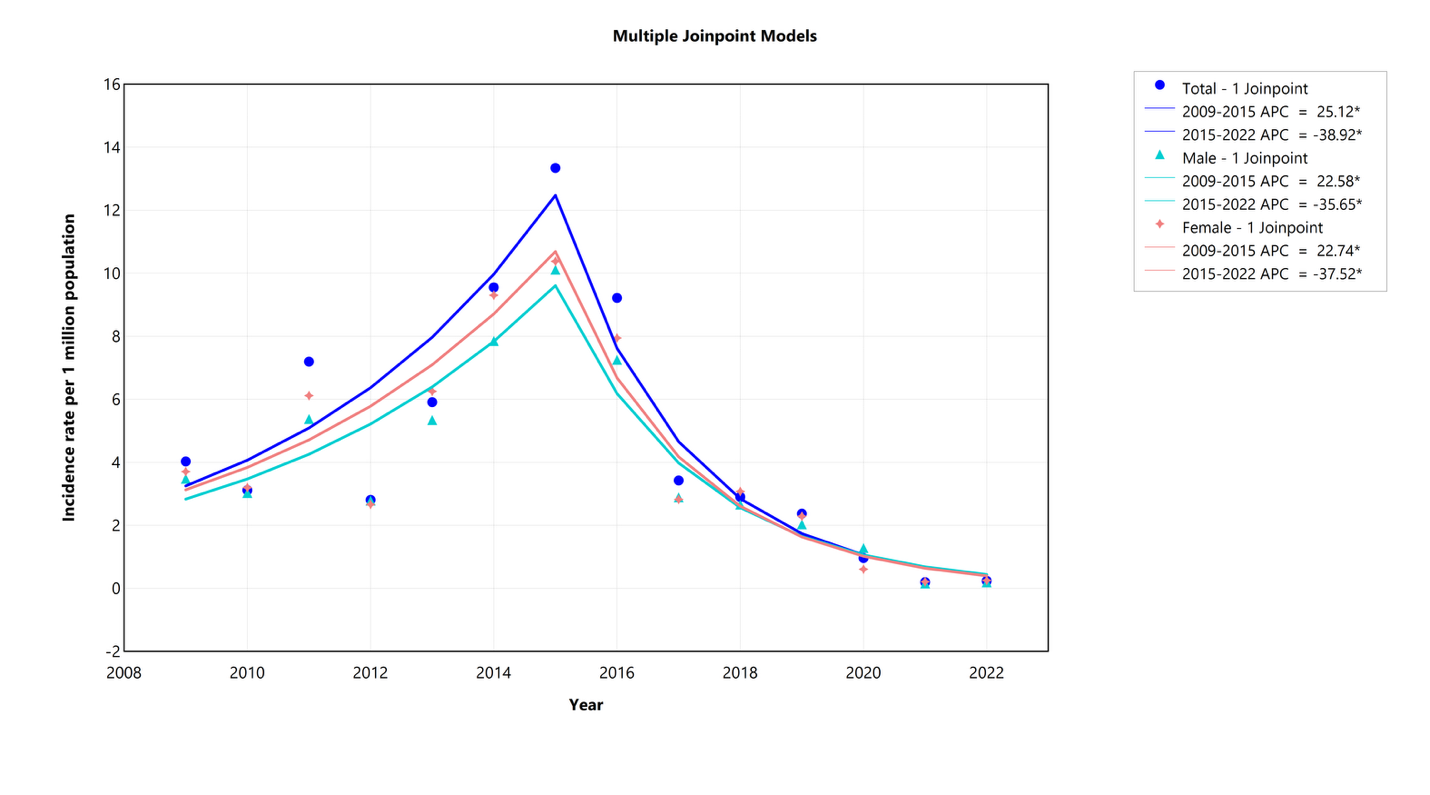


Figure S2. Join-point trends in *Shigella* outbreak-associated case incidence rate by sex groups in the United States, 2009–2022.


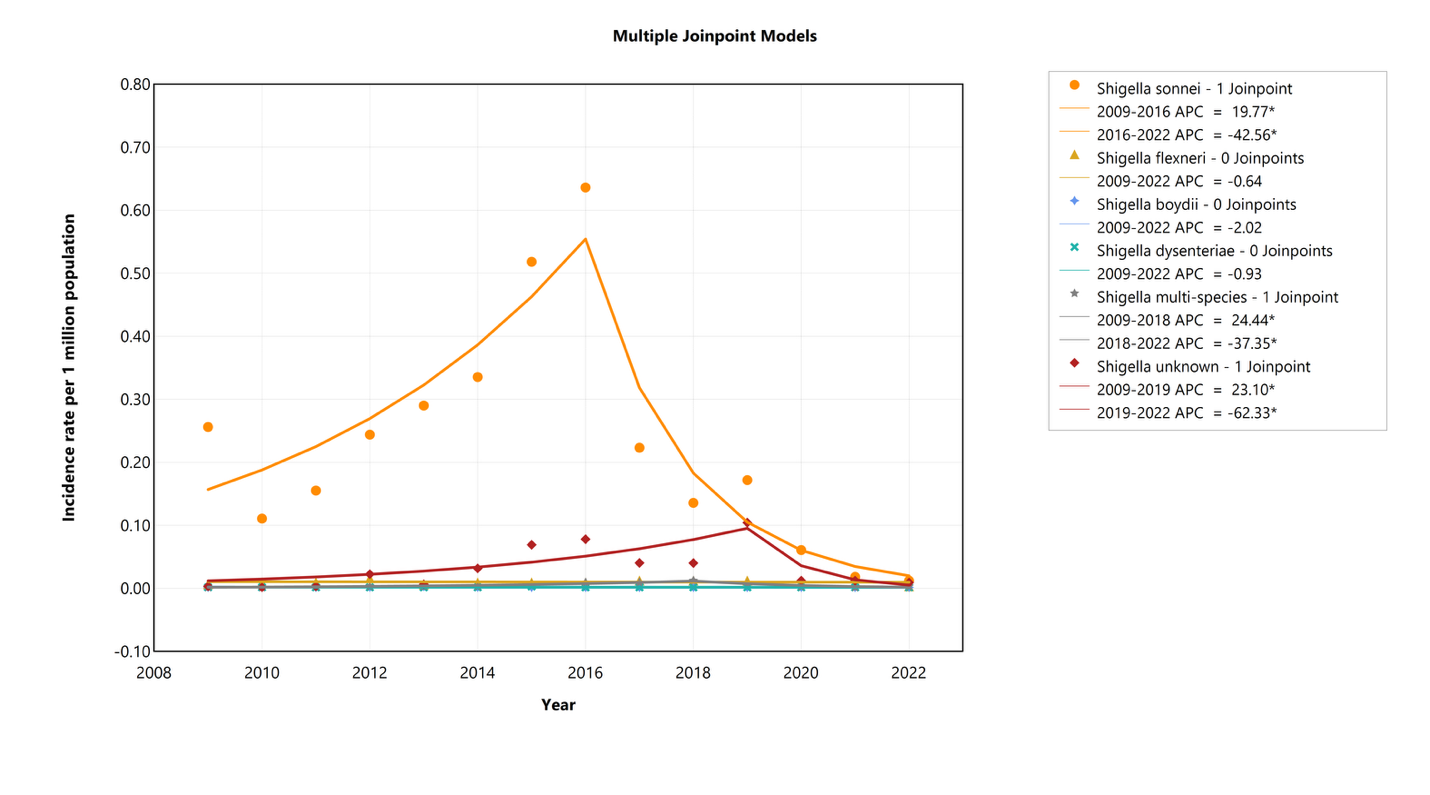


Figure S3. Join-point trends in *Shigella* outbreak incidence rates by species in the United States, 2009–2022.


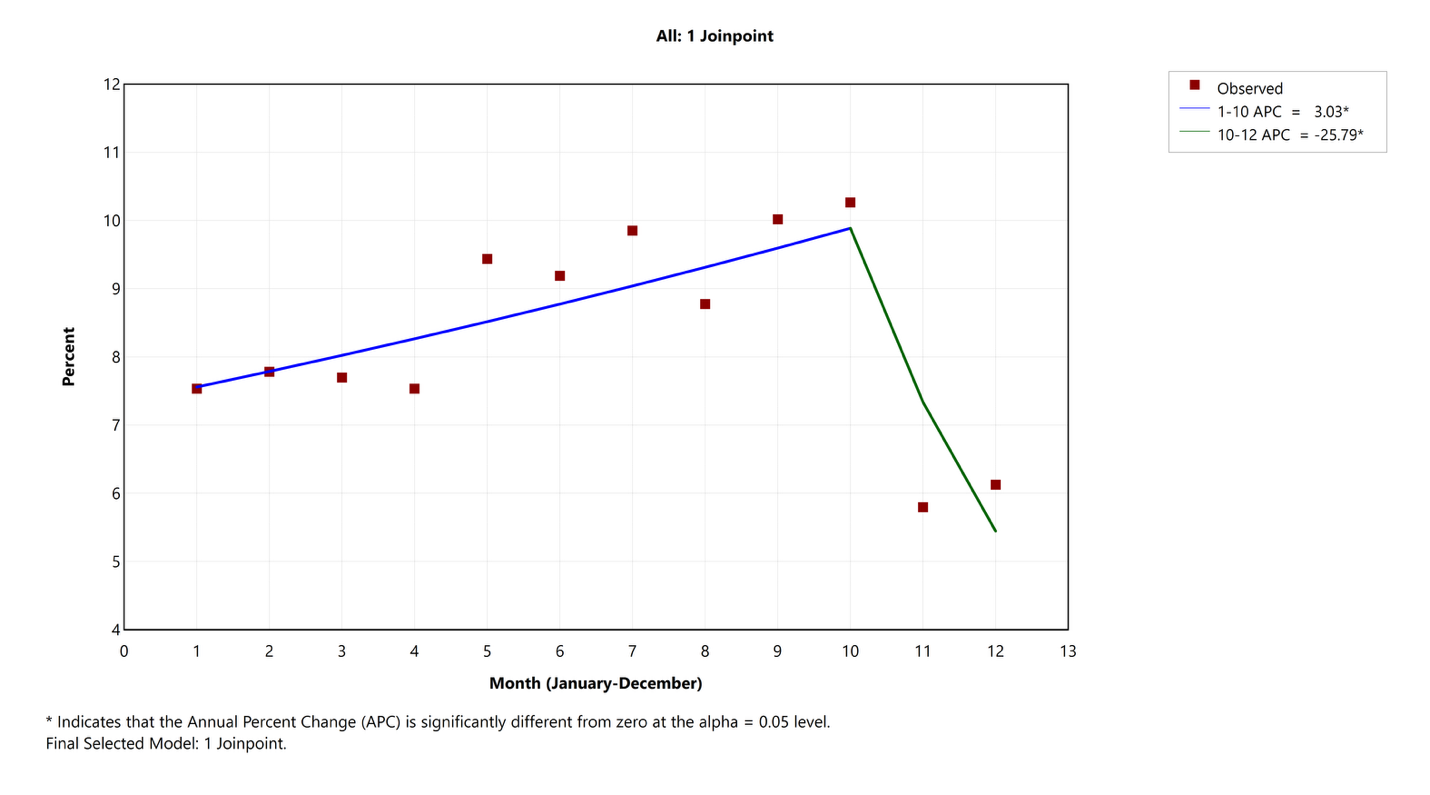


Figure S4. Join-point seasonality trends in *Shigella* outbreaks in the United States, 2009–2022.


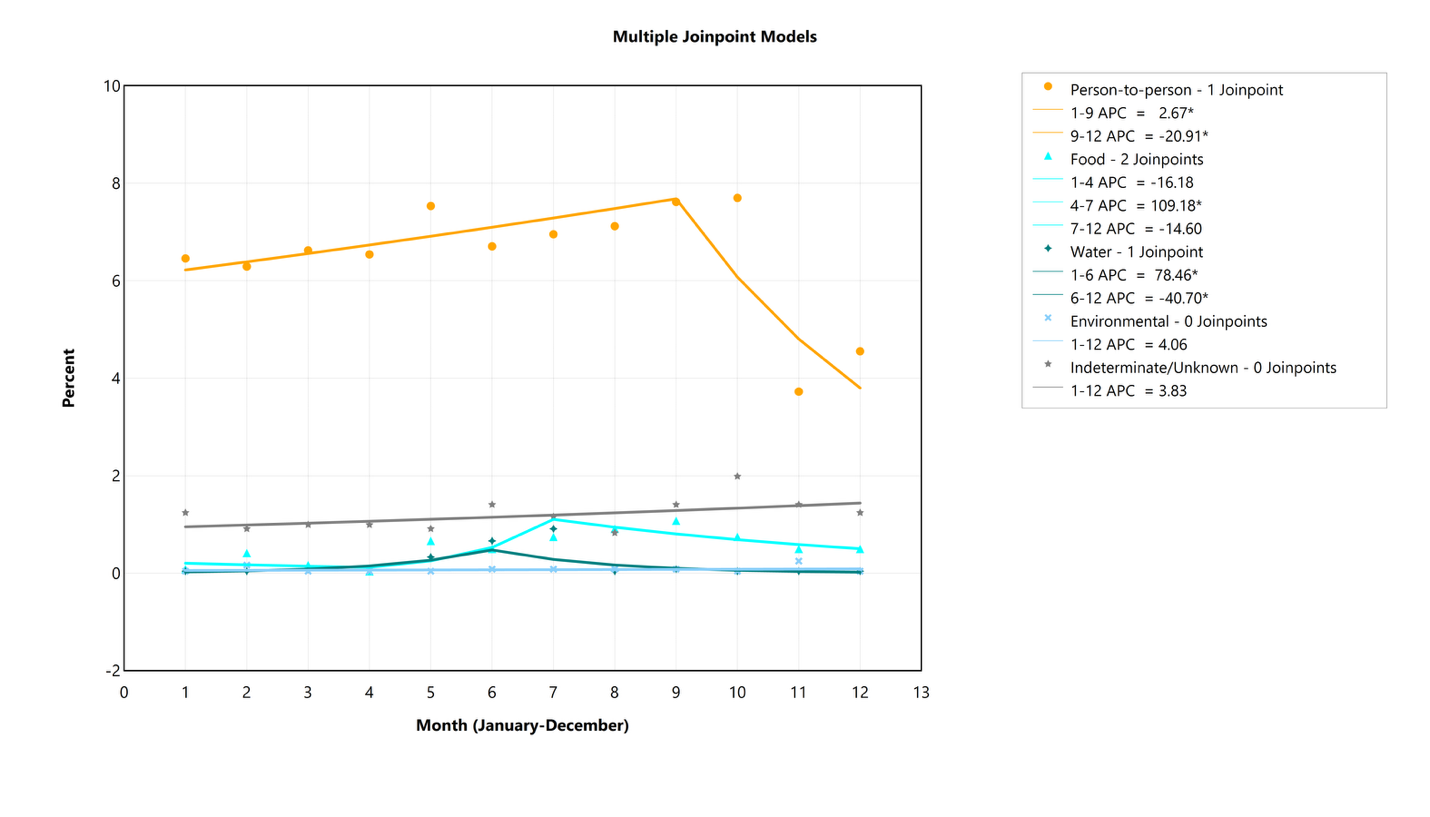


Figure S5. Join-point seasonality trends in *Shigella* outbreaks by transmission mode in the United States, 2009–2022.


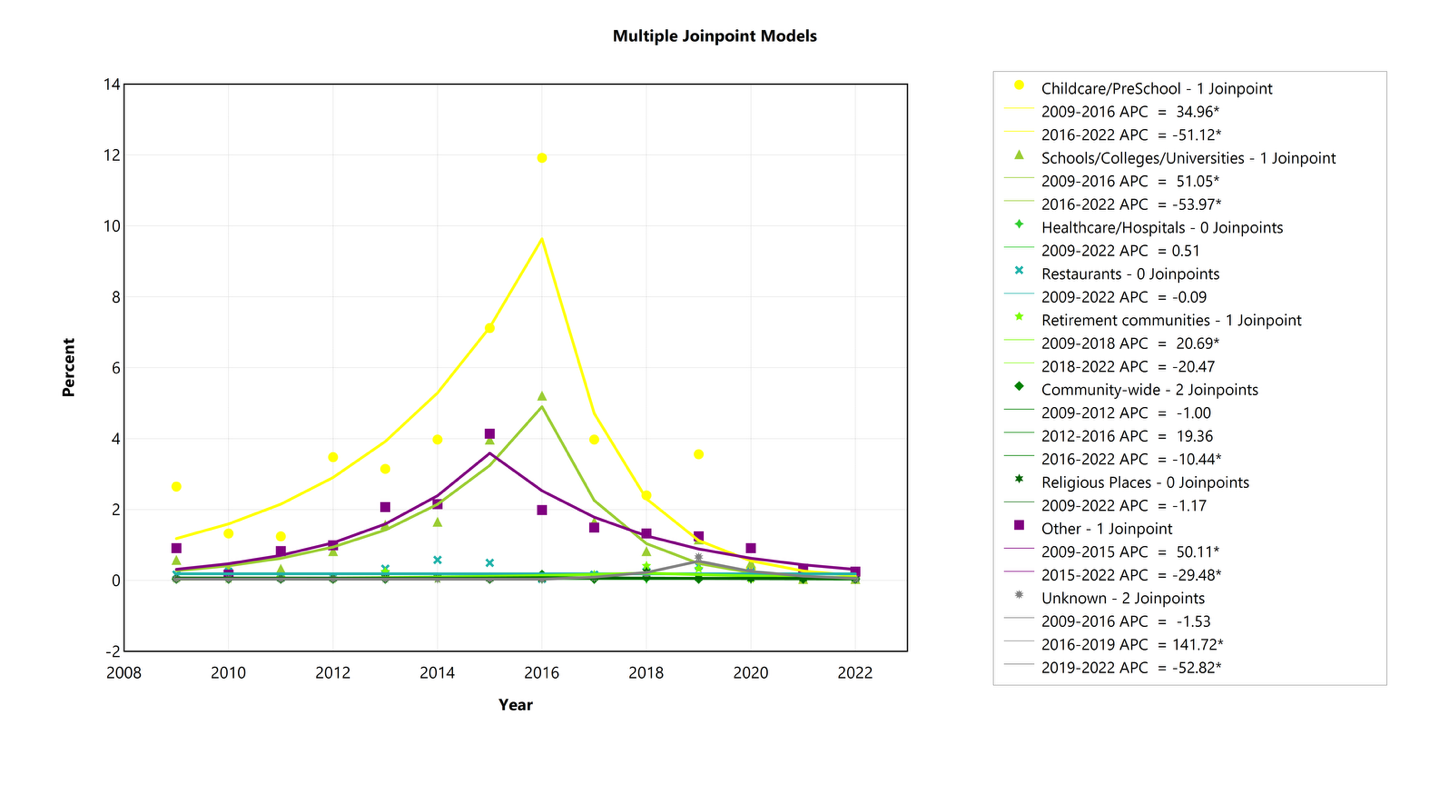


Figure S6. Joinpoint trends in *Shigella* outbreaks by exposure settings in the United States, 2009–2022.


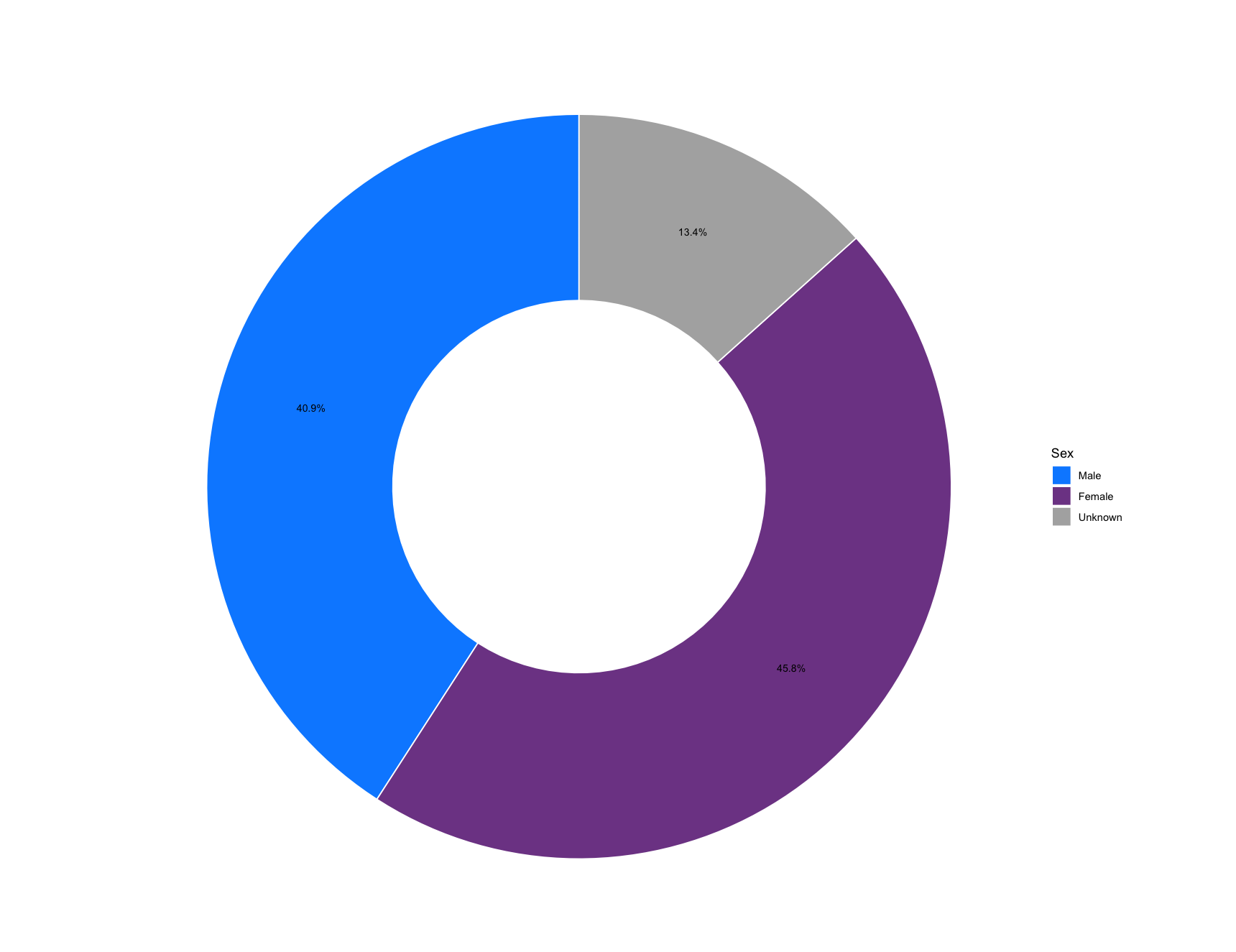


Figure S7. Proportion of *Shigella* outbreak cases by sex group in the United States, 2009–2022.


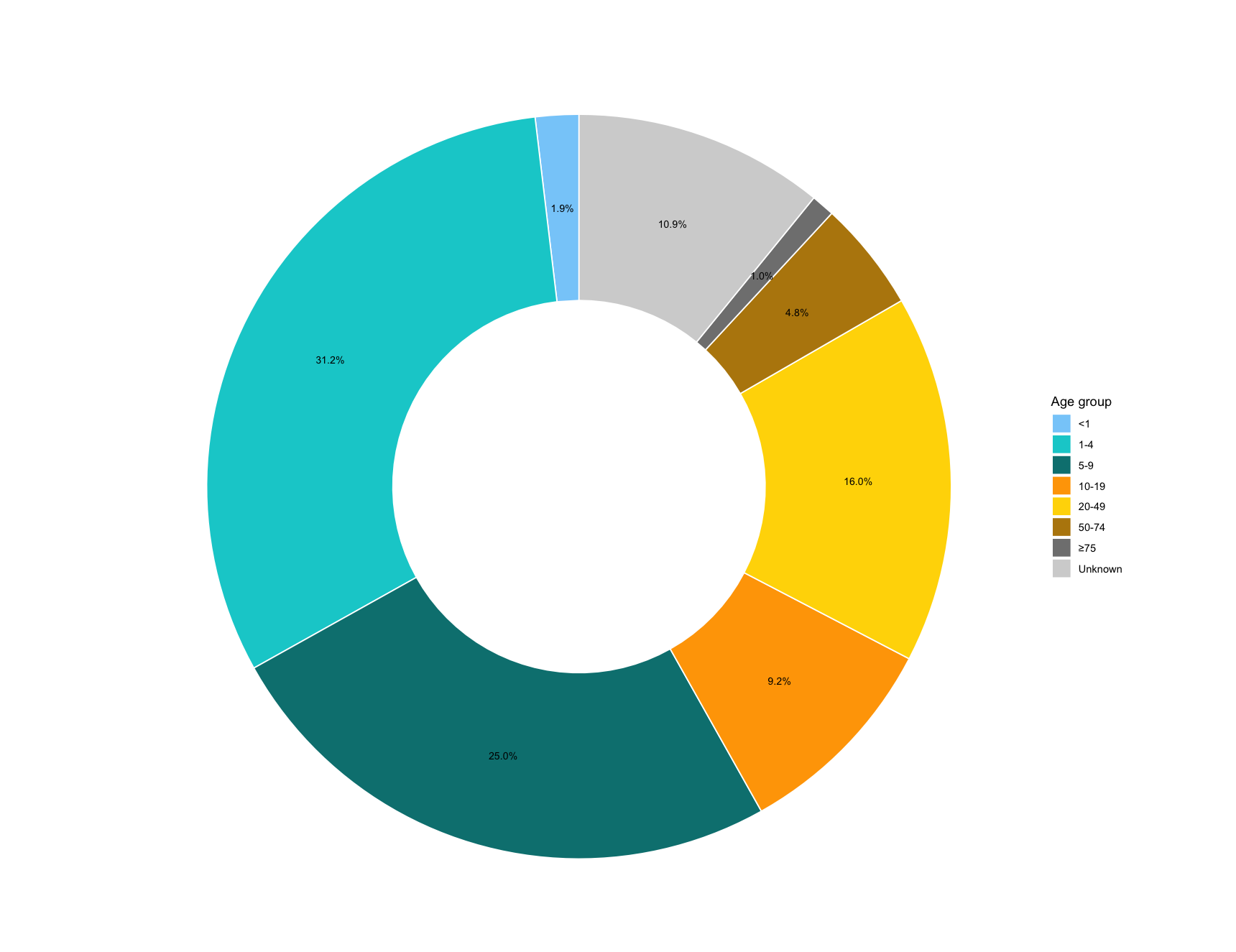


Figure S7. Proportion of *Shigella* outbreak cases by age group in the United States, 2009–2022.
